# The logic layout of the TOL network of *Pseudomonas putida *pWW0 plasmid stems from a metabolic amplifier motif (MAM) that optimizes biodegradation of *m-*xylene

**DOI:** 10.1186/1752-0509-5-191

**Published:** 2011-11-11

**Authors:** Rafael Silva-Rocha, Hidde de Jong, Javier Tamames, Víctor de Lorenzo

**Affiliations:** 1 Systems Biology Program, Centro Nacional de Biotecnología CSIC Cantoblanco-Madrid, 28049 Spain; 2 Institut National de Recherche en Informatique et en Automatique, INRIA Grenoble - Rhône-Alpes, Montbonnot, France

**Keywords:** Regulatory networks, logic gates, TOL network, logicome

## Abstract

**Background:**

The genetic network of the TOL plasmid pWW0 of the soil bacterium *Pseudomonas putida *mt-2 for catabolism of *m-*xylene is an archetypal model for environmental biodegradation of aromatic pollutants. Although nearly every metabolic and transcriptional component of this regulatory system is known to an extraordinary molecular detail, the complexity of its architecture is still perplexing. To gain an insight into the inner layout of this network a logic model of the TOL system was implemented, simulated and experimentally validated. This analysis made sense of the specific regulatory topology out on the basis of an unprecedented network motif around which the entire genetic circuit for *m-*xylene catabolism gravitates.

**Results:**

The most salient feature of the whole TOL regulatory network is the control exerted by two distinct but still intertwined regulators (XylR and XylS) on expression of two separated catabolic operons (*upper *and *lower*) for catabolism of *m*-xylene. Following model reduction, a minimal modular circuit composed by five basic variables appeared to suffice for fully describing the operation of the entire system. *In silico *simulation of the effect of various perturbations were compared with experimental data in which specific portions of the network were activated with selected inducers: *m-*xylene, *o-*xylene, 3-methylbenzylalcohol and 3-methylbenzoate. The results accredited the ability of the model to faithfully describe network dynamics. This analysis revealed that the entire regulatory structure of the TOL system enables the action an unprecedented metabolic amplifier motif (MAM). This motif synchronizes expression of the *upper *and *lower *portions of a very long metabolic system when cells face the head pathway substrate, *m-*xylene.

**Conclusion:**

Logic modeling of the TOL circuit accounted for the intricate regulatory topology of this otherwise simple metabolic device. The found MAM appears to ensure a simultaneous expression of the *upper *and *lower *segments of the *m-*xylene catabolic route that would be difficult to bring about with a standard substrate-responsive single promoter. Furthermore, it is plausible that the MAM helps to avoid biochemical conflicts between competing plasmid-encoded and chromosomally-encoded pathways in this bacterium.

## Background

Prokaryotic regulatory networks are organized in a hierarchical way, on top of which a few transcriptional factors (TF) may coordinate the expression of hundreds of genes of different functional categories (including other downstream TFs), thus linking extracellular conditions to distinct physiological states [1]. It is generally accepted that cell-wide regulatory and metabolic circuits acquire an optimum of performance by connecting a large number of discrete network motifs [2] that, once merged, endow cells with a remarkable ability to deal with changing physicochemical and nutritional scenarios. [3]. In environmental bacteria, such a regulatory optimum is often unsettled following the knock-in of new functions through horizontal gene transfer (HGT), typically by conjugative plasmids [4]. This is because the new encoded traits must find a suitable functional and physical site in the recipient cells to secure their establishment in the new host [5], a process that is not devoid of regulatory, metabolic and structural problems [6]. Many conjugative plasmids of bacteria thriving in sites polluted by recalcitrant chemicals (e.g. compounds released by urban and industrial activity) determine autonomous catabolic systems for biodegradation of such unusual carbon sources [7]. These mobile elements quickly spread through the microbial population of the site upon occurrence of a suitable environmental pressure [8-10]. This creates a natural scenario of network perturbation, as the enzymes and the regulators encoded by both the indigenous genome and the acquired plasmids can interfere with each other. Yet, the literature contains numerous cases of bacteria whose native metabolic complement has been stably expanded to degrade recalcitrant and xenobiotic compounds because of naturally gained catabolic plasmids [11-14]. In these instances, one can safely assume that network implantation conflicts caused by HGT have been ultimately solved. Moreover, the structure of such successful regulatory circuits is likely to bear both the problem and the solution somehow encrypted in their topology and their dynamics.

**Figure 1:**
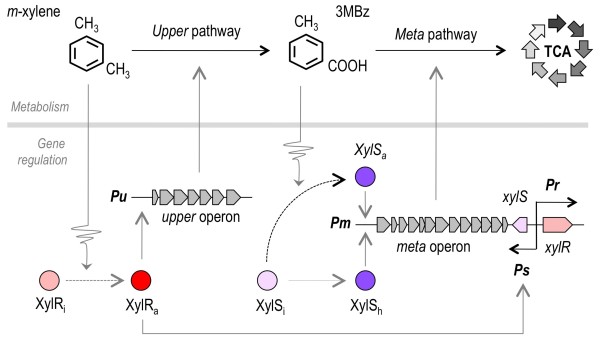
**Overview of the TOL network**. At the metabolic level, *m*-xylene is first converted to 3-methylbenzoate (3 MBz) through the action of the enzymes encoded by the *upper *operon, and this intermediate compound is further metabolized into the TCA cycle by the activities born by the *meta *operon. In the sketch, XylR and XylS are transcriptional regulators while *Pu*, *Pm*, *Ps *and *Pr *are promoters. At the regulatory level, the master regulatory gene *xylR *is encoded in a location adjacent to the end of the *meta *operon and expressed from the *Pr *promoter (not to scale). The corresponding TF is produced in the so-called inactive form (XylRi). This protein changes to an active form (XylRa) when bound to the inducer *m*-xylene or its first intermediate 3-methylbenzyl alcohol (3 MBA, not shown). XylRa then activates both *Pu *and *Ps*, which triggers expression of the *upper *pathway and stimulates production of XylS respectively [50]. In the absence of *m-*xylene, this second regulator XylS is produced at low levels, and it changes from the inactive form XylSi to the transcriptionally proficient XylSa by binding to 3 MBz [23]. This XylSa form is able to induce *meta *pathway expression by activating *Pm*. But, concomitantly, high levels of XylS triggered by XylRa-mediated *Ps *activation can also induce *Pm *activity. This activation loop is formalized as an alternative XylS form (XylSh, for hyper-expressed XylS [32])

The metabolic network encoded in the so-called TOL plasmid pWW0 for *m-*xylene biodegradation carried by the soil bacterium *Pseudomonas putida *mt-2 [15] provides a suitable system to examine the evolutionary interplay between a pre-existing metabolic/regulatory chassis and a novel set of implanted genes that encode extra enzymatic functions. While the plasmid-less strain is able to grow on benzoate through the products of the chromosomally encoded *ben *and *cat *gene clusters [16], acquisition of pWW0 expands the metabolic capacity towards toluene, *m-*xylene and methyl-benzoates (Figure 1). This is brought about by the action of two large plasmid-encoded gene clusters. The so-called *upper *operon encodes enzymes for conversion of *m-*xylene to the corresponding carboxylic acid, (*m-*toluate), while the lower operon takes these products down to central metabolic intermediates: pyruvate and acetaldehyde. The regulation of this system involves two transcriptional factors XylR and XylS that not only separately respond to *m-*xylene and *m-*toluate, respectively, but they are also intertwined in two unusual ways. First, expression of XylS depends on activation of XylR by *m-*xylene. Second, overproduction of XylS suffices to activate the *lower *operon promoter *Pm *even in the absence of its cognate effector, *m-*toluate. As a consequence, the head substrate of the system can activate directly the *Pu *promoter of the *upper *pathway and, indirectly, the *lower *operon as well. As shown in Figure 1 this originates a complex regulatory architecture [17] for controlling what otherwise appears to be a set of simple biochemical transformations. The question at stake is why such a complexity is necessary and what types of regulatory duties are encrypted in it. In other words, what is the rationale for such particular network layout. It is remarkable that such a question has hardly been raised before despite the abundance of molecular details on each of the components of the TOL pathway [15,17]. The study of the system in its entirety has been difficult so far by the lack of a suitable model to examine the behavior of the network as a whole rather than its separate parts. Fortunately, current computational tools allow the dissection of the logic structure of intricate regulatory networks on the basis of their topology, even if many interaction parameters between their constituents remain unknown [18,19]. Boolean formalisms are particularly suited to this end, because adoption of binary logic gates for describing regulatory actions provides a rigorous representation of the system as a decision-making device or *logicome *[3,17]. Furthermore, logic gates grant a mathematical relationship between the interacting components that can be translated into a set of equations for simulating the dynamics of the system [19]. The resulting modularization of the network allows adding complexity by connecting it to new logic gates, as well as network minimization (i.e. model reduction), where unnecessary interactions can be removed in order to generate more compact (and workable) models [20].

We have previously formalized the regulatory network of the TOL system as a digital circuit by converting all known molecular interactions into binary logic operations [17]. In this work, we have further exploited such a Boolean approach for decoding the underlying reason for the complex genetic circuit that controls *m-*xylene metabolism in this plasmid. To this end, we have [i] minimized the TOL logicome by removing non-critical connections, [ii] translated the resulting logic network into a set of piecewise-linear differential equations [21] amenable to a dynamic modeling, [iii] performed simulations on the extant circuit along with counterparts lacking distinct interactions and [iv] matched *in silico *predictions to *in vivo *assays. As shown below, a minimized logicome model is composed of only five variables that not only faithfully described the behavior of the TOL system but revealed that the entire network architecture frames the action of an unprecedented regulatory device that accounts of the entire topology of the system.

## Results and Discussion

### Minimization and streamlining of the catabolic TOL network

The organization TOL regulatory and metabolic circuit of *P. putida *mt-2 for biodegradation of *m-*xylene is shown in Figure 1. The two pathways/operons encoded in the self-transmissible plasmid pWW0 present in this strain are coordinately expressed in response to the aromatic compounds which can be used by this bacterium as a sole carbon source if no other more palatable growth substrate is available [15]. Degradation of *m*-xylene takes place through two series of biotransformations. First, the *upper *pathway encodes enzymes for the conversion of *m*-xylene into *m-*toluate (i.e. 3-methylbenzoate, 3 MBz), which are expressed from the *Pu *upon activation by the regulatory protein XylR in response to the aromatic substrate ([22]; Figure 1). Second, the *meta *(also called *lower*) pathway encodes activities for the ensuing metabolism of 3 MBz into intermediates of the TCA cycle. This second operon of the system is activated by another plasmid-encoded regulator, XylS. This factor has the ability to trigger transcription at the cognate promoter *Pm *either by itself (provided that there is enough concentration of the protein) or in combination with 3 MBz, in which case much lower levels of XylS are required to the same end [23,24]. Apart of these plasmid-encoded regulatory components, a number of host factors (such as σ^70^, σ^54^, σ^38^, σ^32^, IHF and HU) and global regulators (Crc, PtsN, TurA, PprA, ppGpp; [25]) mediate a fine tuning of the system to a large number of environmental signals. Under the same physiological conditions, these default connections to the growth status of the host remain unaffected and they can be basically ignored. In particular, the action of the Crc factor that inhibits XylR translation when cells grow in a rich medium [26] can be suppressed experimentally by culturing cells in a synthetic mineral medium devoid of amino acids and other repressive substrates [27,28].

**Figure 2:**
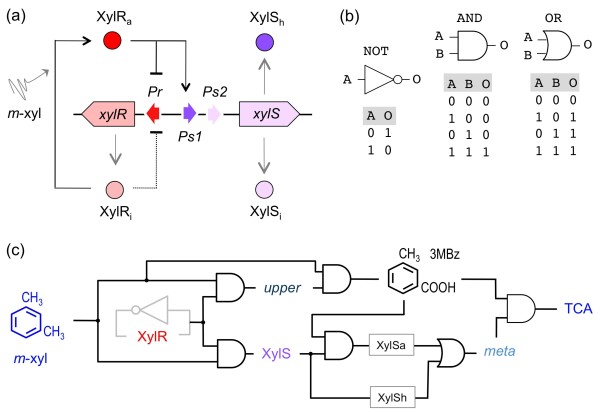
**Formalization the TOL network as a logic circuit**. **(a) **Interplay between transcriptional factors. The scheme blows up the divergent *Pr/Ps *region that controls expression of XylR and XylS, respectively. In the absence of *m-*xylene, XylR represses weakly its own transcription from *Pr*, and an inactive form of XylS (XylSi) is expressed through a low-constitutive divergent promoter *Ps2*. The presence of *m-*xylene both increases XylR auto-repression and activates the σ^54^-dependent *Ps1 *promoter, thereby strengthening XylS expression to the point of reaching a high concentration (XylSh) able to activate the *Pm *promoter of the lower operon (see text). **(b) **Basic logic gates (AND, OR and NOT) used for constructing the model presented in this work, along with the respective truth tables. **(c) **The minimal TOL *logicome*, which represents the core logic interactions taking place in the system. Expression of the *upper *pathway is represented by an AND gate having both XylR and *m*-xylene as inputs, the same being true for XylS production. 3 MBz synthesis is represented also as an AND gate with the *upper *pathway and *m*-xylene as the inputs. For expression of *meta*, the formation of XylSa (XylS plus 3 MBz) is presented as an AND gate where the output is connected to an OR gate, where the second input is overproduced XylS itself (XylSh). This is because *Pm *can be induced by either low level-XylS along with 3 MBz as an effector or high level, effector-free XylS (see text). Finally, degradation of 3 MBz into TCA metabolic intermediate is represented by an AND gate with the *meta *enzymes and the 3 MBz substrate as inputs. Note that the XylR auto-repression loop has been eliminated for the model, since the actual levels of this protein are known to change little in the presence/absence of *m-*xylene.

Figure 1 shows only the TOL-specific regulatory and metabolic constituents of the TOL system, around which the work presented below revolves. Furthermore, the mechanism for regulation of the two TF encoded in pWW0 is of special interest. *xylR *and *xylS *genes are divergently transcribed in a fashion that affords XylR to repress its own transcription (both in the presence or absence of *m-*xylene) as well as activating *xylS *expression in response to *m*-xylene [29] as shown in Figure 2a. This regulatory node makes the system ultimately dependent on changes in XylR levels or activity [27,30]. As described elsewhere [17], known pair-wise interactions between such constituents can be faithfully recreated with Boolean formalisms to produce a logic circuit composed of a number of logic gates (Figure 2b). The resulting relational chart represents the minimal logic structure of the system (i.e. the *logicome*) as a single circuit with defined inputs and outputs (Figure 2c). On this basis, the corresponding ensemble of logic gates (Figure 2c) could then be formalized into a set of piecewise-linear (PL) differential equations. These portray approximate kinetic behaviors by describing the regulation of the synthesis and degradation of proteins and other molecular species by means of Boolean functions (see Methods section and [19]).

### Structure of the minimalist logic circuit that governs the TOL system

As shown in Figure 1, the *upper *TOL pathway is expressed from the *Pu *promoter that is in turn activated by XylR bound to *m*-xylene [15]. As both the XylR protein and the inducer are necessary to trigger *Pu *expression, expression of the *upper *operon has an AND logic (Figure 2b) where both inputs are strictly necessary to generate an output (Figure 2c). In the case of *xylS *expression, its logic is the same as for the *upper *expression as this regulator is expressed from a promoter called *Ps1 *which also requires XylR bound to *m*-xylene for activity [29,31]. Unlike *Pu*, however, a second weak constitutive promoter (*Ps2*) originates a low amount of XylS protein [32]. For *meta *pathway expression, the logic operation performed by the *Pm *promoter (which controls transcription the *meta *operon) is not trivial -in fact it is remarkably puzzling. *Pm *can be turned on by either XylS bound to 3 MBz [33] or by overproduction of the same effector-free XylS due to activation of the *Ps1 *promoter by XylR [23,29]. In this way, *Pm *activation by XylS/3 MBz is represented by a AND gate which is in turn integrated in an OR gate, where the second input is XylS itself (Figure 2c). However, 3 MBz-dependent XylS activation is specified in the PL model as the result of *upper *and *m*-xylene (thus symbolizing the enzymatic degradation of *m*-xylene, see Methods section). This is a reasonable simplification [34] because enzymatic reactions are way faster than expression of the corresponding genes. Therefore, the process of 3 MBz production can be considered instantaneous in terms of time-scale once the enzymes are formed. Assuming such time-scale criteria helps to raise models that are easier to handle and avoid aberrant results likely to occur in qualitative modeling if different temporal hierarchies are mixed up. Another simplification involves XylR. This protein controls negatively its own expression and it is therefore represented by a single NOT gate (Figure 2c). However, since various experiments indicate that XylR auto-regulation allows a constant supply of protein levels [30,35] we formalized XylR expression in the model as a simple unregulated process. The resulting set of equations (Table 1) where instrumental for assembling the *mini-logicome *of Figure 2c, which was employed to inspect the behavior of the TOL network under a number of operational conditions.

**Table 1:** Equations and threshold inequalities used to simulate the TOL network

N°	PL equations for the TOL model	Description
**1**	dupper/dt = k^0^_upper _* s^+ ^(XylR, θ_XylR_) * s^+ ^(m_xyl_, θ_mxyl_) - g_upper _* upper	*Upper pathway expression*
**2**	dXylS/dt = k^0^_XylS _+ k^1^_XylS _* s^+ ^(XylR, θ_XylR_) * s^+ ^(m_xyl_, θ_mxyl_) - g_XylS _* XylS	*XylS expression*
**3**	dmeta/dt = k^0^_meta _* s^+ ^(XylS, θ^2^_XylSh_) + k^1^_meta _* s^+ ^(XylS, θ^1^_XylSi_) * s^+ ^(upper, θ_upper_) * s^+ ^(m_xyl_, θ_mxyl_) - g_meta _* meta	*Meta pathway expression*
**4**	dXylR/dt = k^0^_XylR _- g_XylR _* XylR	*XylR expression*

	**Parameter inequalities**	

	*zero_upper _<θ_upper _< k^0^_upper_/g_upper _< max_upper_*	*Parameter inequalities for equation 1*
	*zero_XylS _< θ^1^_XylSi _< k^0^_XylS_/g_XylS _< θ^2^_XylSh _< (k^0^_XylS _+ k^1^_XylS_)/g_XylS _< max_XylS_*	*Parameter inequalities for equation 2*
	*zero_meta _< θ_meta_< k^0^_meta_/g_meta _< k^1^_meta_/g_meta _< (k^0^_meta _+ k^1^_meta_)/g_meta _< max_meta_*	*Parameter inequalities for equation 3*
	*zero_XylR_<θ_XylR _< k^0^_XylR_/g_XylR _< max_XylR_*	*Parameter inequalities for equation 4*

	**Alternative parameter inequalities**	

	*zero_XylS _< θ^1^_XylSi _< k^0^_XylS_/g_XylS _< (k^0^_XylS _+ k^1^_XylS_)/g_XylS _< θ^2^_XylSh _< max_XylS_*	*No XylS hyper-expression condition (for eq. 2)*
	*zero_upper _< k^0^_upper_/g_upper _<θ_upper _< max_upper_*	*No XylSa condition (for eq. 1)*

### Coarse description of TOL network dynamics

In order to simulate the activation of the TOL network in response to *m*-xylene, equations 1-4 (see Table 1) where implemented in the Genetic Network Analyzer software (GNA; [21]), as described in the Methods section. Also, *m*-xylene was placed as an input variable [21], meaning that [i] no PL equation is specified in the model associated to this component, and [ii] its concentration is not allowed to change during the simulations. As a pre-requisite to perform model simulation, *parameter inequalities *(Table 1) where defined for all variables in the system as described previously [21]. This approach allows setting the thresholds of the interaction processes, a fundamental attribute when a component of the system has more than one target or synthesis rate (which is indeed the case in TOL).

**Figure 3:**
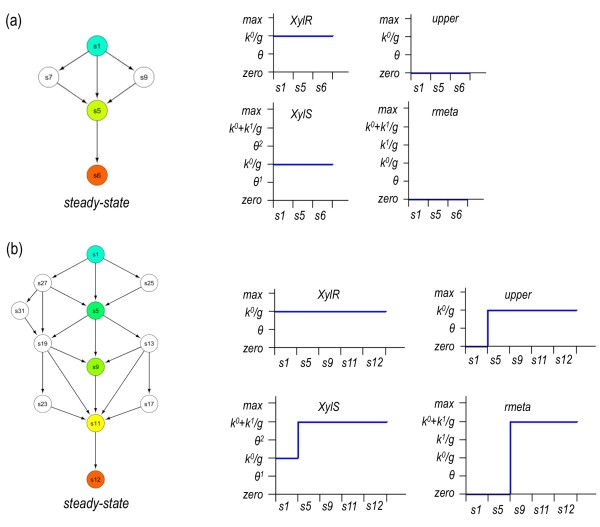
**Simulation of the TOL logicome in the presence or absence of *m*-xylene**. Piecewise-linear differential equations describing the regulatory and metabolic events of the network were implemented in GNA software and the behavior of the TOL simulated in response to *m*-xylene. **(a) **Non-inducing conditions. The state transition graph resulting from the simulation is shown to the left with the shortest path between defined states indicated in color. The plots to the right show the temporal sequence of qualitative states for the two regulators (XylR and XylS) and the two pathways (*upper *and *meta*) in the selected path of the transition graph. **(b) **Induced conditions. The transition graph is shown to the left while the temporal sequence of qualitative states is displayed to the right. As before, color states highlight the shortest path in the transition graph.

A first simulation contemplated the TOL system in the presence of the input *m*-xylene or its absence. In either condition, a single steady-state was found in the transition graph generated (Figure 3). A state transition graph describes the qualitative dynamics of the network, indicating the possible states of the system (concentration levels of enzymes and regulators) and transitions between these states occurring under the influence of regulatory events. It should be noted that the states of a transition graph (annotated with an *s *letter followed by a number) do not signify time intervals but occurrence of consecutive conditions regardless of the time it takes to move from one state to the other. For instance, shift from *s1 *→ *s5 *in Figure 3a happens before *s5 *→ *s6 *but it says nothing on the time involved in each transition: numbers 1, 5 and 6 refer to the name of the state but not to any temporal scale. A *steady state *characteristically lacks a successive stage in the transition graph. If *m*-xylene is low, only XylR is high (i.e. above the threshold defined in Table 1), while XylS is at low level (i.e. below that necessary for activating *Pm *in the absence of 3 MBz, see Table 1) and the *upper *and *meta *levels/activities are zero (Figure 3a). When *m*-xylene is high, all elements are high at steady-state. Inspection of the transition graph (which represents the successive order of events) in *m*-xylene-high condition shows a progression of regulatory steps identical to the known activation itinerary of the TOL pathway i.e. both *upper *and XylS are expressed in response to XylR activation, followed then by activation of *meta *due both to 3 MBz formation and XylS hyperexpression (Figure 3b). This coarse equivalence between the non-perturbed Boolean model and the recognizable behavior of the system *in vivo *in response to *m-*xylene set a reference for inspecting *in silico *the inner network logic as explained below.

### XylR is the master regulator of a synchronized single-input module (SIM)

**Figure 4:**
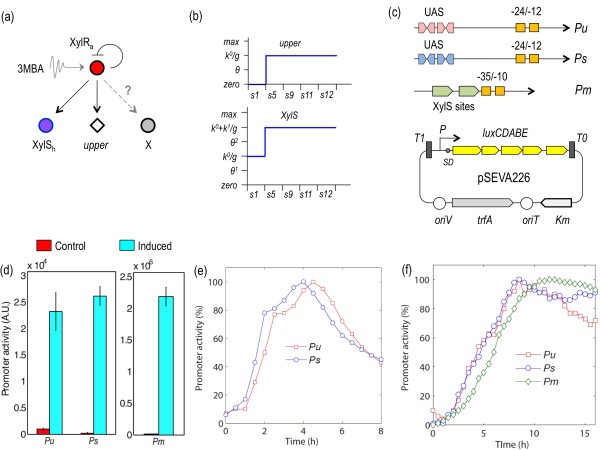
**Analysis of of *Pu *and *Ps *activation dynamics by XylRa**. **(a) **Proposed single-input module for XylR (SIM_XylR_). In this motif, XylRa controls negatively its own expression and activates XylS and the *upper *pathway. While no other target is known for XylR, it cannot be excluded that this regulator controls additional genes (represented as X). **(b) **Simulations for *upper *and *xylS *expression under inducing condition show the synchrony of gene activation. **(c) **Genetic constructs used to analyze promoter kinetics. The architecture of *Pu*, *Ps *and *Pm *are sketched. The UAS (for upstream activator sequences) for XylR in *Pu *and *Ps*, and the XylS binding sites of *Pm *are shown, with an indication of the boxes for σ^54^-RNAP (-12/-24) and σ^70^-RNAP (-10/-35) recognition. Below, the main features of the broad host range *lux *reporter vector pSEVA226 were each of the promoters was cloned are indicated. **(d) **Light emission of reporter strains *P. putida *mt-2 (pSEVA226*Pu*), *P. putida *mt-2 (pSEVA226*Ps*) and *P. putida *mt-2 (pSEVA226*Pm*). Each of the strains was cultured in minimal medium with succinate and then added with 5 mM 3MBA as described in the Methods section. Light emission was recorded after 4 h and the figures of bioluminescence/OD_600 _converted into arbitrary promoter activity units, A.U. **(e) **Induction kinetics of *Pu *and *Ps *assayed in minimal/succinate medium and 1 mM of 3MBA. Reporter strains *P. putida *mt-2 (pSEVA226*Pu*) and *P. putida *mt-2 (pSEVA226*Ps*) were treated as before but the aromatic inducer was present throughout the entire growth. **(f) **Relative induction kinetics of *Pu*, *Ps and Pm *in minimal medium with 5.0 mM 3MBA as the sole carbon source. Promoter activities were normalized in all cases in respect to their respective maximum values. Note the virtual identity between *Pu *and *Ps *promoters and the delay of *Pm*.

The chief control step for TOL expression relies on signal (toluene/*m*-xylene) sensing by XylR. As mentioned before, this regulator of the NtrC family targets two σ^54^-dependent promoters, *Pu *and *Ps1 *(Figure 1). This network motif, where a master regulator controls multiple targets in response to a single signal is named *single-input module *(SIM, Figure 4a) and it is overrepresented in bacterial regulatory networks [2]. The basic property of a SIM motif is that by having different affinities for multiple targets, the master regulator can impose a temporal order of gene expression in response to the same signal [2,36]. This *just in time *gene expression device is often used for the control of genes encoding a complex machinery (e.g., the flagellum) where the components must be assembled in a given order [37]. In the case of the TOL system, the temporal order of *Pu *vs. *Ps *activation is foreseen to have considerable consequences for the system, because XylR-dependent XylS hyper-expression is transmitted downstream into the node controlling *meta *expression (Figure 1).

In order to examine the consequences of having XylR as the upstream regulator in the SIM_XylR _motif we simulated the response *Pu *and *Ps *under various *θ_XylR _*parameter values (Table 1). To this end we just varied the value of the *θ_XylR _*parameter in equations for *Pu *and *Ps*. The same value for the two promoters means synchronization (i.e. XylR is equally capable to activate *Pu *and *Ps*; Figure 4b) whereas setting different *θ_XylR _*parameters for each promoter results in a temporal order of activation (not shown). But what is the actual state of affairs in the TOL system *in vivo*? To answer this question we analyzed experimentally the activation kinetics of *Pu *and *Ps*. For this, we cloned these promoters upstream of a promoter-less *luxCDABE *operon placed in a low-copy broad-host range plasmid (Figure 4c). The resulting transcriptional fusions were introduced into a wild type *P. putida *mt-2 strain to faithfully monitor the dynamics of the TOL system. The system was induced with 3-methylbenzyl alcohol (3MBA) as a proxy of *m-*xylene. 3MBA is the first intermediate of the biodegradation route and it is equally able to trigger the TOL system [15]. Furthermore, its much higher solubility (in contrast to the volatile *m-*xylene) makes 3MBA more suitable for induction experiments in liquid media [38]. As shown in Figure 4d, both cloned promoters were efficiently induced upon 3MBA exposure. In order to quantify the response of *Pu *and *Ps *to 3MBA, overnight grown cells were diluted in fresh M9 medium supplemented with either 3MBA as the sole carbon source or with 3MBA plus succinate. The luminescent signals of the strains were quantified along the growth curve and normalized respect to the respective optical density at 600 nm. The very small offsetting between *Pu *and *Ps *observed in the medium with both succinate and 3MBA (Figure 4e) disappeared altogether in the culture where 3MB was employed as sole C-source (Figure 4f). The behavior of both promoters is thus virtually identical under the conditions tested (absolute values were also comparable, not shown). The lack of significant differences in the timing or overall kinetics of *Pu *and *Ps *activation indicated that the SIM_XylR _motif of the TOL network operates in a synchronous way for triggering expression of the *upper *pathway and the *xylS *gene. Finally, we could observe that *Pm *activity reached its maximum activity with a noticeable delay in respect to *Pu *and *Ps *(Figure 4f), as anticipated with the results of the simulation of Figure 3b. This delay is expected because *Pm *functionality does require more steps (production of XylS, formation of 3 MBz) than the instant trigger of *Pu *and *Ps *by effector-activated XylR (XylRa).

The results above were very informative because -to the best of our knowledge- synchronous SIM motifs have not been reported before in genetic networks. The role of SIM_XylR _for the TOL circuit dynamics is therefore likely to be crucial. If *upper *were expressed earlier than *xylS*, 3 MBz production would occur also earlier than maximal expression of *meta *(i.e. *Pm *activation by hyper-expressed XylSh would be delayed) and it would thus result in a transient accumulation of 3 MBz. In contrast, if *xylS *were activated before *upper*, expression of *meta *would start earlier and cells would have the degradation machinery for 3 MBz in place before the compound could actually materialize from *m-*xylene biodegradation. Interestingly, proteins TurA and PprA have been recently demonstrated to interfere with XylR binding to the *Pu *promoter but not to *Ps *[39,40]. Such interference, which is factually equivalent to decreasing the affinity of XylR for *Pu*, would favor the second scenario (i.e. *meta *expressed before 3 MBz appears), thereby suggesting that these proteins have a role to set a temporal order in activation of the TOL operons. Alas, the signals that trigger TurA and PprA activities are unknown [39,40].

### Expression of the *meta *operon reflects the combination of two separate activation loops

**Figure 5:**
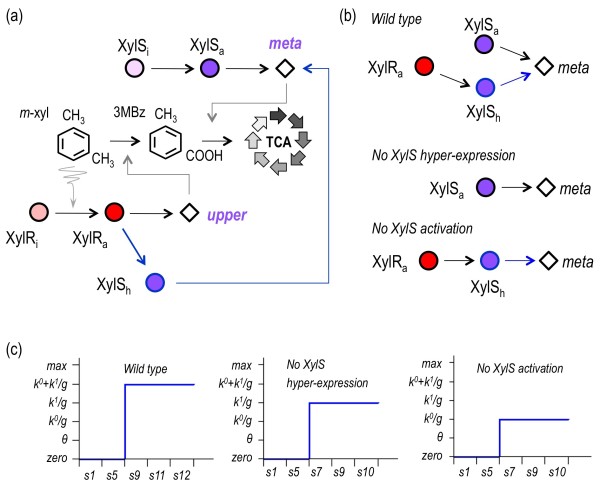
**Modeling the effect of the XylSh loop in TOL system**. **(a) **Signal transmission/conversion in the TOL system. The diagram sketches interactions between the active forms of the regulators and the metabolic intermediate 3-methylbenzoate (3 MBz). To the left, inactive XylR (XylRi) becomes activated by *m-*xylene to produce the transcriptionally competent form XylRa. This in turn, results in activation of the *upper *pathway and overproduction of XylS (XylSh), which can by itself activate *meta *pathway. Such a XylSh loop (marked in blue), which does not involve 3 MBz, links the *meta *pathway directly to *m*-xylene presence. To the right, XylS produced at low levels, insufficient for activating *meta *(XylSi) turns into an active form (XylSa) to the same end upon binding the 3 MBz produced by the action of *upper *on *m-*xylene. Finally, production of *meta *converts 3 MBz into Krebs' cycle intermediates. **(b) **Simulation conditions. *Wild type *considers the complete model where *meta *is concomitantly expressed through both XylSa-mediated and XylSh-mediated paths. In *No XylS hyper-expressed *conditions the effect of XylSh has been removed and *meta *is activated only by XylSa. In *No XylS activation *condition, the effect of XylSa has been deleted and *meta *is under the sole control of XylSh. **(c) **Temporal sequence of qualitative states for each of the three conditions. Each scenario was simulated until the system reached a steady state.

As mentioned above, expression of *Pm/meta *takes place through an uncommon process, in which the same regulator (XylS) operates *either *by itself at high concentrations *or *bound to 3 MBz, albeit at lower protein concentrations [24]. This is formalized by operatively considering 3 forms of the protein: inactive (XylSi), active by binding the inducer (XylSa) and active by hyperproduction (XylSh). This last protein form (which may not be a different protein species, but XylSi at high concentrations) causes what we call the XylSh loop, that links production of the *meta *pathway directly to the first input of the system *m-*xylene (Figure 5a). The logic of the circuit tells us that kinetics of this XylSh loop must intrinsically rule the timing of expression of the *meta vs*. the *upper *pathway, thereby accounting for the fine temporal tuning of *Pm *output. To clarify the significance of such a dual regulation of *Pm *by XylSa and XylSh we entered their absence/presence as variables of the TOL model. We implemented such *in silico *mutation by changing *parameter inequalities *for XylRa-dependent XylS expression and for the capability of high levels of XylS (i.e. XylSh) to activate *Pm *(see Table 1 and Methods section). These relatively small modifications were enough to inspect the behavior of the TOL network as shown in Figure 5b. The new *parameter inequalities *were implemented separately and the result of the simulations were compared to the wild-type network. The results shown in Figure 5c indicated that the expression levels of the *meta *pathway are lower in each separate TOL variant while the timing of *Pm *activation remains the same than the intact system.

**Figure 6:**
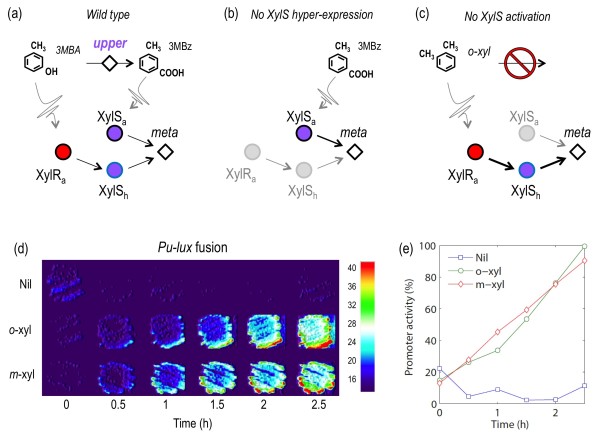
**Experimental strategy for quantification of XylSa-dependent and XylSh-dependent *Pm/meta *activation**. **(a) **Default scenario, i.e. *Pm *is activated by both XylSa and XylSh. The inducer employed in this case is *m*-xylene (or its proxy 3MBA), which both activates XylR (and thus triggers the XylSh loop and is metabolized by *upper *to produce 3 MBz, necessary for XylSa formation. **(b) **XylSa alone i.e. no XylSh. The added inducer is 3 MBz, which is specific for XylS. **(c) **XylSh alone i.e., no XylSa. The inducer employed is *ortho-*xylene (*o*-xylene), which fully activates XylR (thus generating high levels of XylS = XylSh) but cannot be converted into 3 MBz and therefore XylSa cannot be formed. **(d) ***Pu *activation by *m*-xylene and *o*-xylene. Reporter strain *P. putida *mt-2 (pSEVA226*Pu*) was patched on the surface of minimal-succinate agar plates, grown overnight and then exposed to saturating vapors of either inducer as indicated. Bioluminescence was captured along time and the figures in arbitrary units represented with a color code according to the signal intensity (bar on the right represents the scale). Nil: Control with no inducer. **(e) **Promoter activities on the basis of the densitometry of the images of panel (d). Values were normalized in respect to maximum activity as above.

The predictions above were tested with *in vivo *experiments in which we analyzed the induction kinetics of the *Pm *promoter using again the complete *luxCDABE *operon as reporter system. For this, a wild type strain of *P. putida *mt-2 harboring a plasmid with the *Pm-luxCDABE *fusion at stake was separately exposed to different inducers known to have distinct effects in the rest of the network. In one case, grown cells were exposed to *m*-xylene (formally equivalent to 3MBA employed before). This inducer not only does activate XylR (thereby triggering the XylSh loop) but it also makes *m*-xylene to be converted into 3 MBz through the action of the *upper *pathway, which leads to formation of XylSa (Figure 6a). In sum, *m-*xylene/3MBA originate both XylSh and XylSa. In a second case, the one inducer that was exogenously added was 3 MBz, which coverts XylS only into XylSa, i.e. the XylSh loop is not activated. Finally, *Pm- luxCDABE *cells were exposed to *o-*xylene. This is as good inducer of XylR as *m*-xylene, but it is not a substrate of the *upper *pathway [41] and thus cannot be converted into 3 MBz. This makes effector-less, overproduced XylSh the only possible activator of *Pm*. This simple choice of inducers causes a selective action of XylSa, XylSh or both on *Pm*, what faithfully mimics the effect of lacking each of these components of the circuit in the simulations above. Note that -given the volatile nature of *m-*xylene and *o-*xylene, we resorted to an *in situ*, non-disruptive method to record the response of the *Pm-luxCDABE *cells to these inducers. For this, the strains under examination were streaked in small sectors of M9-succinate plates and let grown overnight at 30°C. The plates with the patches of bacterial growth were then exposed to saturating vapors or *m*-xylene or *o*-xylene and the luminescent signals recorded every 30 min with a photon-counting device. To have a control that *m*-xylene and its non-metabolizable analogue *o*-xylene were equally able to trigger XylR activation under such experimental conditions, a *Pu-luxCDABE *fusion strain was subject to the same procedure as well.

**Figure 7:**
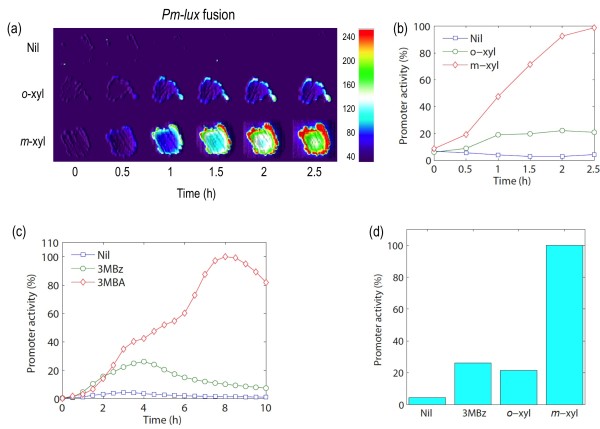
***Pm *regulation through alternative control loops**. **(a) ***Pm *activation in response to vapors of *m*-xylene or *o*-xylene assayed in solid media. Patches of the reporter strain *P. putida *mt-2 (pSEVA226*Pm*) were grown on the surface of M9-succinate agar and then exposed to saturating vapors of *m-*xylene (which triggers the appearance of both XylSh and XylSa), or *o*-xylene (which makes cells to produce only XylSh). **(b) ***Pm *promoter activity deduced from images of panel (a) processed identically as in Fig. 6. **(c) ***Pm *activation kinetics in liquid media added with 1 mM 3MBA (*m-*xylene proxy, leading to both XylSh and XylSa) or 1 mM of 3 MBz (appearance of XylSa only). Promoter activities of reporter strain *P. putida *mt-2 (pSEVA226*Pm*) are shown in respect to the maximal value reached with 3MBA induction. **(d) **Contribution of each regulatory device to *Pm *activity. The bar diagram compares standardized promoter activities brought about by the XylSa-dependent loop (3 MBz), by the XylS hyper-expression loop (*o*-xylene induced) and both (*m*-xylene or 3MBA induced). Promoter activity is represented as the maximal value obtained in every experimental condition relative to the highest *m*-xylene (or 3MBA) induction value.

As shown in Figure 6d, the expression profiles of *Pu *in response to both xylene species were virtually identical, as quantification of the signal intensities in both conditions gave nearly overlapping induction curves (Figure 6e). In contrast, the effect of each of these aromatics on the strain with the *Pm-luxCDABE *fusion was different, as *o*-xylene triggered a lower response than the metabolizable inducer. Quantification of signal intensities revealed that *o*-xylene-mediated *Pm *stimulation was ~20% of that brought about by *m*-xylene (Figure 7b). This figure reports the relative contribution of XylSh to *Pm *functioning but it does not tell us much about the XylSa-only counterpart. To tackle this, we examined the response of *Pm *to 3 MBz (i.e. caused only by XylSa) *vs*. the sum of XylSa and XylSh made happen by 3MBA. In this case, *Pm-luxCDABE P. putida *cells were inoculated in liquid M9/succinate medium, added with 1.0 mM 3 MBz or 1.0 mM 3MBA and the promoter activities monitored along the growth curve (see Methods). As shown in Figure 7c, maximal *Pm *induction by 3 MBz was ~25% of the expression level of the promoter in cells exposed to 3MBA, a percentage close to the same contribution of the XylSh-only loop.

The outcome of these *in vivo *experiments is that the *Pm *induction levels derived from each of the two forms of XylS are similar when acting separately but they become synergistic by >4-fold when working together (Figure 7d). This is mechanistically easy to explain, because overproduced XylSh can be converted to XylSa by exposure to 3 MBz. The XylSh loop thus ensures [i] that expression of the *meta *pathway is well underway before 3 MBz is formed through the action of the *upper *TOL pathway and [ii] that the lower route is boosted very significantly by 3 MBz. These *in vivo *results not only match the findings stemming from model simulations discussed above but also suggest that the rationale of the regulatory architecture of the TOL network is to maintain a good level of all products of the two operons at all times following exposure to *m-*xylene and thus avoid any transient accumulation of 3 MBz by first anticipating its production from *m-*xylene (the XylSh loop) and then by amplifying expression of the 3 MBz-degrading genes (i.e. the lower pathway) as soon as 3 MBz is formed. This regulatory device could have evolved to solve a metabolic conflict between the enzymatic modules encoded in the TOL plasmid and the indigenous metabolic network of the host, as argued below.

## Conclusions

**Figure 8:**
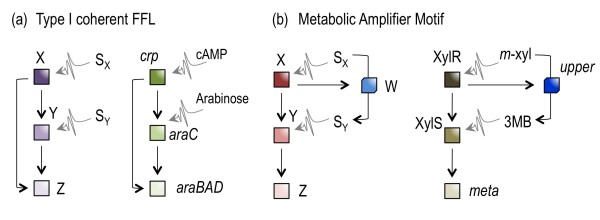
**The inner logic of the TOL regulatory network**. **(a) **Layout of a canonical type I coherent Feed Forward Loop (FFL). In such a motif, a master regulator × activates expression of a target Z both directly and indirectly. Indirect regulation takes through activation of a second transcriptional factor Y which in turn has Z as a target as well. S_X _and S_Y _are the signals which trigger the activity of × and Y, respectively. The *ara *operon is shown as an example of this type of FFL, as its expression depends on the interplay between the CRP and AraC regulators, cAMP and arabinose being the S_X _and S_Y _inducers respectively. **(b) **Metabolic Amplifier Motif (MAM) found in the TOL network. Compared to the type-I FFL motif, the indirect regulation of Z through the X→Y node remains, but the direct interaction X→Y makes a detour that involves a metabolic (rather than regulatory) action. Specifically, the master regulator × now activates the production of an enzyme (or a metabolic pathway) W, which converts the signal S_X _into S_Y_. In the TOL system, × and Y are represented by XylR and XylS, while *m*-xylene (S_X_) is converted to 3 MBz (S_Y_) by the action of the *upper *pathway.

The dual activation mode of *Pm *by XylS is intriguing as it resembles, but does not entirely match, the feed forward loop (FFL) motif frequently found in regulatory networks [2,42]. In a typical FFL motif, a master transcription factor (TF) controls a gene Z directly and also regulates a second TF, which in turn has Z as a target (Figure 8a; [43]). Depending on the sign of the interaction between these components (i.e. negative or positive) as well as the logic of signal integration at the target Z (e.g. AND or OR), the resulting FFL endows the circuit with different properties e.g. filtering transient changes in the input signal or delay in the ON/OFF responses [43]. In the TOL system just examined, the forward interplay X→Y→Z (XylRa → XylSh →*meta*) does occur, but × does not interact directly with Z (i.e. XylR does not activate directly *meta*). Instead, XylR controls the expression of a metabolic conversion component (the *upper *pathway) that translates the upstream input (*m*-xylene) into the downstream input 3 MBz, which in turns enhances expression of *meta *(Figure 8b). The TOL circuit has thus properties reminiscent of those of coherent type-I FFLs with an OR logic, as intermediate expression levels were experimentally observed for the target Z (*meta*; [43]). However, we argue that this mode of operation is not just one variant of FFL, but a new network motif in itself. In fact, the interactions just described can be formalized as a distinct regulatory pattern composed of 2 intertwined regulators X→Y (in our case, XylR→XylS), 2 cognate effector molecules S_X _(*m-*xylene) and S_Y _(3 MBz) and one metabolic activity W triggered by the first regulator that converts one effector into the other (S_X_→S_Y_). This arrangement (which we have designated *metabolic amplifier motif *or MAM, Figure 8b) not only ensures a good expression of catabolic system involving the consecutive action of many different genes. It also presets the TOL system to deal with the appearance of 3 MBz and accelerates removal of this compound once it is formed. Metabolic anticipation/amplification is clearly the effect of such a MAM. But there might be an added bonus to this scenario, because undue accumulation of 3 MBz may lead to its non-productive misrouting into the chromosomal benzoate-biodegradation pathway of *P. putida *[41] and generation of toxic dead-end intermediates [44]. The MAM reported here may thus have helped to maintain the TOL pathway as an autonomous metabolic machinery that interacts only minimally with the central carbon consumption routes of the host cells.

## Methods

### Mathematical modeling with piecewise-linear differential equations

Formalization of each of the regulatory components of the TOL system (Figure 1) as binary logic gates and the assembly of the complete network as a digital circuit has been described before [17]. The streamlined scheme of Figure 2c was used as a guide to the formulation of equations for each of the regulatory and metabolic steps of the network. To this end, we adopted piecewise-linear (PL) differential equations [45] for describing the circuit dynamics as described [21,46]. The rationale of this approach is that production of metabolites in the model is determined by state equations, which define the metabolic reactions as the result of [i] the presence of substrates and [ii] the activation of a *regulation function *that encodes transcriptional and metabolic interactions. Under this scheme, a regulatory function accounts for the expression of a given gene and the production of the corresponding protein owing to the presence of an effector. Specific regulatory functions can thus be expressed as:

(1) $$f_{i}\left(x_{j}\right)=k_{i}*s^{+}\left(x_{j},\Theta_{j}\right)$$

This equation indicates that the synthesis of product *i *is a function of the presence of the effector *j*. In this equation, *s*+ is a *step function*, a Boolean operator that is set to a value 1 if the concentration of *j *(*x_j_*) is above a particular threshold (*θ_j_*), and to a value 0 if the concentration is below *θ_j_*. The synthesis of *i *occurs at a rate determined by *k_i _*only when *x_j _*>*θ_j_*, and does not take place if *x_j _*<*θ_j_*. In this case, *j *is an activator of the synthesis of *i*. By the same token, the effect of a repressor can be represented using a negative step function:

(2) $$f_{i}\left(x_{j}\right)=k_{i}*s^{-}\left(x_{j},\Theta_{j}\right)$$

which is set to a value 1 when *x_j _*<*θ_j_*, and to a value 0 when *x_j _*>*θ_j_*. On this basis, the production of a given component of the network as a result of the combined influence of different effectors can be written as, e.g.:

(3) $$f_{i}(x_{j},x_{k})=k_{i}*[s^{+}(x_{j},\Theta_{j})*s^{-}(x_{k},\Theta_{k})]$$

where the appearance of product *i *is regulated by the activator *j *and the repressor *k*. Such a component *i *is synthesized only if *j *is present and *k *is absent. This is equivalent to a logic device (a logic gate) involving an AND/NOT operator (ANDN), taking *j *and *k *as inputs and producing *i *as output. In this way, it is possible to model all possible transcriptional and metabolic interactions in the system as logic constructs, determining the production of particular compounds as a function of the presence or absence of some of the others [47]. Furthermore, it is possible to set different thresholds for the concentration of a particular compound when it controls different synthesis rates at different concentrations. For instance, if effector *j *regulates the synthesis of A and B, we can set the constraint *θ_j_^1 ^*<*θ_j_^2^*, to indicate that synthesis of A is regulated by a lower concentration of *j *than the synthesis of B. These constraints are known as *threshold inequalities *[21] and are described below for the TOL system. Such *threshold inequalities *are related to the effective concentration of a given molecular species (e.g., a TF) above which it is able to have a regulatory effect on a target (e.g. promoter). In the case of TF-promoter pairs, inequalities are entered in the corresponding equations by means of a threshold value (*θ_j_*) which represents the relative affinity of the TF under consideration for one or more target promoters. The active concentration of each of the components is then determined by its production rate (expressed by *k_i_*), and its degradation rate (g_i _* x_i_), which is a strictly positive function, proportional to the concentration of the compound. These simple expressions of positive and negative step functions were adopted for representing all possible regulatory interactions in the system. The resulting set of equations was then implemented with the GNA (Genetic Network Analyzer) software [21]. GNA models describe the evolution of the regulatory circuit by specifying qualitative constraints on the parameters of the system. This allows the model to be reliably run even if the actual threshold concentrations and the reaction rates at stake are not known. One set of constraints thus includes such *threshold inequalities*. A second type of constraints consists of the so-called *focal inequalities *that set the possible steady-state concentrations of the components in the system with respect to their threshold values, for instance:

(4) $$zero<{\Theta_{i}}^{1}<k_{i}∕g_{i}<{\Theta_{j}}^{2}<max$$

This inequality indicates that when component *i *is produced at rate *k_i _*and degraded with a rate constant *g_i_*, so that its concentration converges towards the level *k_i_/g_i _*[45], it exceeds the threshold *θ_i_^1^*, but not *θ_i_^2^*. This allows an estimate of the concentration of the components in reference to their threshold values, even in absence of quantitative information on the parameters of the system. The inequalities for the TOL model were set as shown in Table 1.

It is possible to follow the dynamics of the regulatory network by computing a temporal progression of so-called qualitative states, each consisting of the levels of the concentration variables with respect to their thresholds. In each qualitative state the trend of the concentration variables (increasing/decreasing/steady) determines the possible transitions to successor states. The resulting directed graph of qualitative states and transitions between qualitative states is called a *state transition graph *(for a more detailed description, see [21,46]. Note that the PL equations above and the associated transition graph describe the temporal order of signal propagation in the network when the first input signal is present and the system moves toward a steady state (where the concentrations of the components stop to change). The actual time interval during which the system remains in a state before reaching the next is not contained in these qualitative models. However, the representation reliably predicts the temporal ordering of states, and thus the consecutive changes in the levels of each of the components of the network.

Using the biological assumptions for known regulatory interactions (Figure 1) and the resulting logic operations (Figure 2c), we defined four PL equations describing XylR, XylS, *upper *and *meta *production (Table 1). The sole input for the system was *m*-xylene, which was defined as an *input variable *i.e. one having a constant concentration along the simulations, [21]. For implementation of *in silico *mutations, we changed *threshold inequalities *as follows. In one case *Pm *activation was simulated in the absence of the XylSh loop by setting the parameter *θ^2^_XylSh _*to be higher than the maximal concentration reachable upon *Ps *activation (No XylS hyper-expression condition, Table 1). Similarly, simulation of the *Pm *activation event in a scenario lacking XylSa, we set the *upper *pathway not to produce enough concentrations of 3 MBz for creating XylSa (No XylSa condition, Table 1). Consideration of different *threshold inequalities *in the TOL model allowed us to simulate the specific conditions as discussed in the Results section.

#### Strains, chemicals and growth conditions

*E. coli *CC118 strain [48] was used as the host for plasmid constructions and maintenance, while *P. putida *mt-2 [41] was employed for the analysis of promoter activity with reporter constructs (see below). *E. coli *was grown in Luria-Bertani (LB) medium at 37°C. Unless indicated otherwise, *P. putida *was cultured in a minimal medium M9 supplemented with MgSO_4 _(2.0 mM), citrate or succinate (0.2%) as the only carbon source and grown at 30°C. Plasmids were conjugally transferred from *E. coli *to *P. putida *with a tripartite mating procedure [48] using *E. coli *HB101 (RK600) as the helper strain. When required, growth media was supplemented with kanamycin (Km, 50 μg/ml) or chloramphenicol (Cm, 30 m/ml). All chemicals and substrates, including aromatic effectors (*m*-xylene, *o*-xylene, 3-methylbenzyl alcohol and 3-methyl benzoate) were purchased from Sigma-Aldrich.

#### Construction of reporter gene fusions

The TOL promoters *Pu*, *Ps *and *Pm *were separately cloned in pSEVA226, a Km^R ^broad host range vector (*RK2 *origin of replication) bearing a promoterless *luxCDABE *[49] operon downstream of the multiple cloning site of pUC (Silva-Rocha *et al*., in preparation). To this end, each of the promoters of interest was amplified from *P. putida *mt-2 DNA through PCR reactions with Pfu DNA polymerase (Promega) using primer pairs PUF (5'-GCG GAA TTC TTG ATC AAA TC GA CA GG TG GT TAT G-3') and PUR (5'-GCG CGG ATC CGT CTC GTA TAG CTA GCA ACC GCC-3') for *Pu*, PSF (5'-GGC CGA ATT CAT TCC ATC TGC CAC TTT AG-3') and PSR (5'-CGG CCG GAT CCC GGT TCT CTT ATT TTA ATG TGG-3') for *Ps*, and PMF (5'-CGG CCG AAT TCG GTT TGA TAG GGA TAA GTC C-3') and PMR (5'-CGG CCG GAT CCT CTG TTG CAT AAA GCC TAA-3') for *Pm*. These primers introduced in each case *Eco*RI and *Bam*HI sequences in equivalent sites of the 5' and 3' regions of each promoter (underlined in the primer sequence). PCR products were purified, digested with *Eco*RI and *Bam*HI (NewEngland BIolabs), ligated to pSEVA226 cleaved with the same enzymes and transformed in chemically competent *E. coli *CC118 cells. The resulting clones were named pSEVA226-*Pu*, pSEVA226-*Ps *and pSEVA226-*Pm*. Sequence fidelity of the cloned promoters was verified in all cases by DNA sequencing.

### Promoter activity quantification and data processing

The activity of the TOL promoters in response to inducers was examined with different procedures depending on the nature of the specific chemical tested. In case of soluble inducers (3MBA and 3 MBz), overnight grown *P. putida *cells harboring the reporter plasmid under examination were diluted 1:20 in fresh minimal media with the inducer of interest at a final concentration of 1 mM. 200 μl aliquots (with four replicates) of the thereby diluted cells were placed in 96-well Microplates (Optilux™, BD Falcon) and incubated in a Wallac Víctor II 1420 Multilabel Counter (Perkin Elmer) at 30°C, the optical density at 600 nm (OD_600_) and the bioluminescence being recorded every 30 min. Promoter activity was quantified by normalizing bioluminescence in respect to cell density (i.e. bioluminescence/OD_600_). For testing volatile inducers (*m*-xylene and *o*-xylene), single colonies of *P. putida *clones bearing the reporter plasmids indicated were patched on M9/citrate agar plates, grown overnight an exposed to saturating vapors of a 1 M inducer solution in DMSO. Non-disruptive monitoring of promoter output was carried out with a VersaDoc™ Imaging System (BioRad) and the results processed with the ImageJ software (http://rsbweb.nih.gov/ij/). In either case, graphic representations of promoter activities were generated with MATLAB software (MathWorks).

## Authors' contributions

JT and VdL conceived the work, HdJ and RS-R carried out the experiments and the simulations, RS-R and VdL wrote the paper. The authors declare that they have no conflict of interest. All authors read and approved the final manuscript.
